# Model organisms at the heart of regeneration

**DOI:** 10.1242/dmm.040691

**Published:** 2019-10-01

**Authors:** Eleanor L. Price, Joaquim M. Vieira, Paul R. Riley

**Affiliations:** Burdon Sanderson Cardiac Science Centre, Department of Physiology, Anatomy and Genetics, University of Oxford, Oxford OX1 3PT, UK

**Keywords:** Cardiac regeneration, Regenerative medicine, Cardiac repair, Myocardial infarction, Cardiomyocytes

## Abstract

Heart failure is a major cause of death worldwide owing to the inability of the adult human heart to regenerate after a heart attack. However, many vertebrate species are capable of complete cardiac regeneration following injury. In this Review, we discuss the various model organisms of cardiac regeneration, and outline what they have taught us thus far about the cellular and molecular responses essential for optimal cardiac repair. We compare across different species, highlighting evolutionarily conserved mechanisms of regeneration and demonstrating the importance of developmental gene expression programmes, plasticity of the heart and the pathophysiological environment for the regenerative response. Additionally, we discuss how the findings from these studies have led to improvements in cardiac repair in preclinical models such as adult mice and pigs, and discuss the potential to translate these findings into therapeutic approaches for human patients following myocardial infarction.

## Abstract (precis)

**Summary:** This Review discusses the different model organisms used to study cardiac regeneration and their contributions to our understanding of the complex processes that govern regeneration of the heart.

## Introduction

Myocardial infarction (MI), commonly caused by coronary artery occlusion, is a leading cause of mortality worldwide. Improved clinical interventions, including percutaneous coronary intervention (PCI), and the use of drugs to assist the remaining uninjured myocardium, such as angiotensin-converting enzyme (ACE) inhibitors and β-blockers, mean the number of people surviving the initial attack has significantly increased (Arós et al., 2006; Puymirat et al., 2012). However, many still eventually die of end-stage heart failure due to the inability of the adult mammalian heart to replace the billions of cardiomyocytes lost during the MI (Velagaleti et al., 2008). Instead, a non-contractile, fibrotic scar is deposited at the site of injury, leading to functional overload and pathological remodelling, characterised by dilation of the myocardium and eventually heart failure (Pfeffer and Braunwald, 1990). Current therapies manage the symptoms and progression of heart failure but fail to address the damaged muscle (Gheorghiade et al., 2005). At present, heart transplant is the only bona fide cure but, given the issues of immune rejection and lack of donor hearts, there is now an urgent need to therapeutically stimulate regeneration in the adult mammalian heart.

**Fig. 1.**
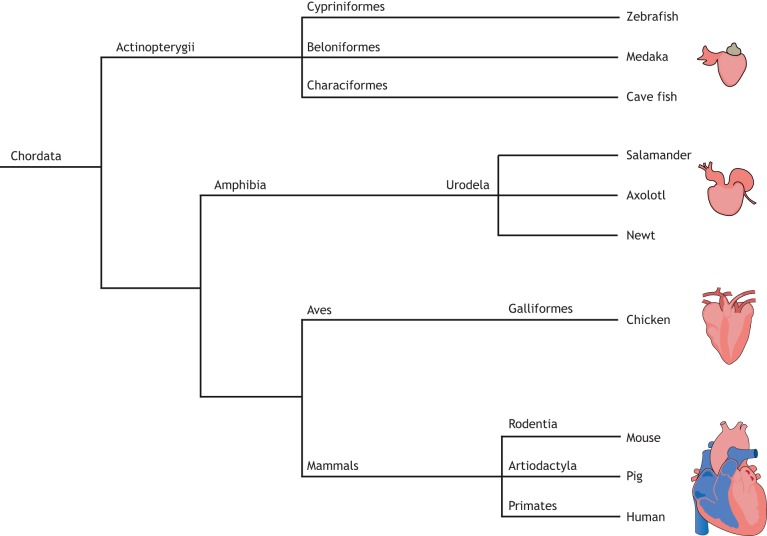
**Animal models of cardiac regeneration.** Phylogenetic tree to demonstrate the evolutionary divergence of the different vertebrate species used in the study of cardiac regeneration.

Whilst the adult human heart has limited regenerative capacity, complete regeneration of damaged tissue and organs is seen in many living organisms across the animal kingdom. Invertebrates such as planarians, echinoderms, annelids and *Hydra*, have global regenerative potential (Candia Carnevali et al., 1998; Dawydoff, 1954; Gates, 1950; Ham and Eakin, 1958; Morgan, 1898), with planarians possessing the capacity to regenerate from as little as ∼1/279 of the original organism (Morgan, 1898). Similarly, some vertebrates, such as newts, axolotls and zebrafish, are capable of regenerating entire limbs or fins following amputation (Pfefferli and Jazwinska, 2015; Simon and Tanaka, 2013), and can regenerate several organs, including the heart, after injury (Cano-Martinez et al., 2010; Chablais et al., 2011; Gonzalez-Rosa et al., 2011; Poss et al., 2002; Witman et al., 2011). Comparative analysis of model organisms of cardiac regeneration provides insight into the evolutionarily conserved molecular mechanisms of regeneration (Fig. 1). This knowledge could enable the reactivation of such processes in the adult human heart following MI.

This Review aims to discuss the various animal models of cardiac regeneration, assessing how they are already contributing to novel therapeutic approaches for heart repair and how they may do so in the future.

## Zebrafish

**Fig. 2.**
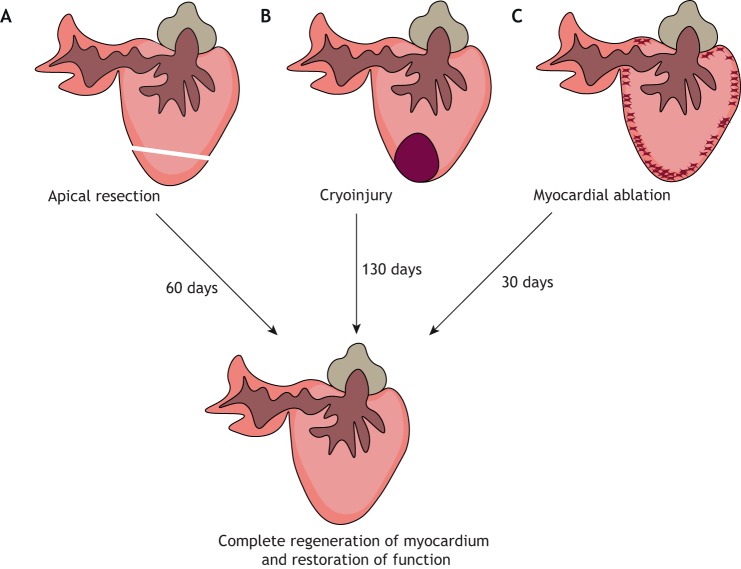
**Methods of cardiac injury in the zebrafish.** (A) Apical resection involves surgical removal of ∼20% of the ventricular apex. (B) In the cryoinjury model, ∼25% of the ventricle is damaged by placing a cryoprobe onto the heart. (C) Genetic ablation leads to the loss of ∼60% of cardiomyocytes.

The zebrafish (*Danio rerio*) is a teleost fish with a two-chambered heart and has been widely studied due to its external development, tissue transparency, genetic amenability and extensive regenerative capabilities. The first definitive description of complete cardiac regeneration in zebrafish was shown in a seminal study by Poss and colleagues in 2002. The authors described complete regeneration of the cardiac muscle, without residual scarring, 60 days after resection of ∼20% of the apex (Fig. 2A) (Poss et al., 2002). Although this represents an interesting model for the study of cardiac regeneration, unlike a human MI this type of injury does not involve ischaemia-induced cell death and the removal of necrotic cell debris. Moreover, resection resulted in the formation of a fibrotic clot, consisting mainly of fibrin fibres, and only minor collagen deposition, which does not resemble mammalian scar formation (Poss et al., 2002).

More recently, a cryoinjury model was established, which involves inflicting localised damage to ∼25% of the ventricle using a cryoprobe (Fig. 2B) (Chablais et al., 2011; Gonzalez-Rosa et al., 2011). This led to massive cell death in the injured area, which was subsequently cleared and replaced by a fibrotic collagen scar, thus more closely recapitulating the set of events that follow a human MI. However, unlike mammals, zebrafish progressively eliminate the scar, replacing it with regenerated myocardium, eventually leading to complete restoration of the damaged tissue, which suggests that scarring does not inhibit cardiomyocyte proliferation and muscle regeneration in this model (Chablais et al., 2011; Gonzalez-Rosa et al., 2011). Significantly, regeneration following cryoinjury is much slower than following ventricular resection, with full functional recovery taking up to ∼180 days, depending on injury size (Hein et al., 2015).

Another zebrafish-based model of heart regeneration involved the genetic ablation of up to 60% of cardiomyocytes (Fig. 2C). Wang and colleagues generated a transgenic line in which a tamoxifen-inducible Cre-recombinase was under the control of the cardiomyocyte-specific promoter *cmcl2*, and expression of the cytotoxic diphtheria toxin A (DTA) was controlled by a *loxP*-flanked STOP cassette. Thus, upon tamoxifen administration, DTA was activated in *cmcl2*-expressing cells, resulting in cardiomyocyte-specific cell death, preservation of endocardial and epicardial tissues, and absence of necrosis or a scar (Wang et al., 2011). Whilst this model is not applicable for deciphering the effects of processes such as cell necrosis and inflammation, it provides a better understanding of the signals that govern cardiomyocyte proliferation, including epicardial- and endocardial-derived retinoic acid (RA) production (Wang et al., 2011).

**Fig. 3.**
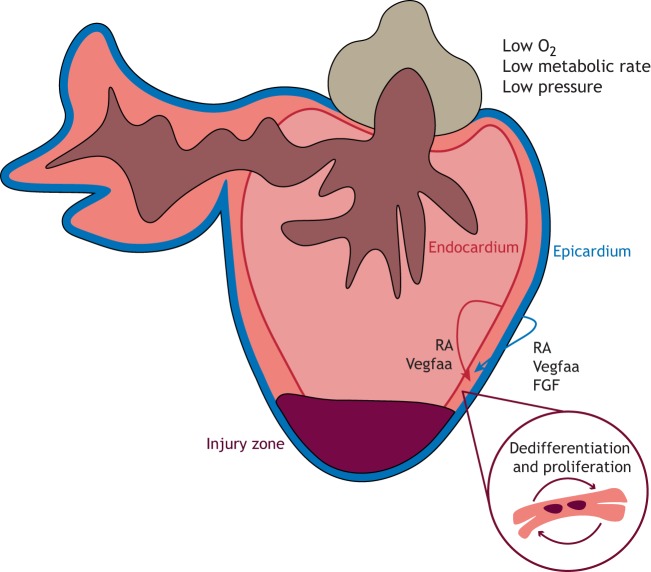
**Cardiac regeneration** **in zebrafish.** Following cardiac injury, diploid cardiomyocytes undergo dedifferentiation and proliferate to replace the damaged tissue. Signals from the activated epicardium and endocardium, such as retinoic acid (RA) and vascular endothelial growth factor Aa (Vegfaa), stimulate such proliferation, whilst epicardial fibroblast growth factor (FGF) signalling induces neovascularisation to restore blood flow.

Whilst these different methods vary in the degree of inflammation and transient scarring, and in the rate of regeneration, they all result in the generation of new muscle, predominantly from the proliferation of pre-existing cardiomyocytes, which undergo de-differentiation and re-enter the cell cycle (Fig. 3) (Gonzalez-Rosa et al., 2011; Jopling et al., 2010; Kikuchi et al., 2010, 2011; Poss et al., 2002).

Mammalian cardiomyocytes are predominantly polyploid, meaning that they contain more than two sets of homologous chromosomes, a characteristic often associated with terminal differentiation (Brodsky et al., 1980; Orr-Weaver, 2015). In contrast, Gonzalez-Rosa and colleagues reported that ∼99% of zebrafish cardiomyocytes are diploid. Further, upon generating a transgenic zebrafish line in which the myocardium was susceptible to polyploidisation, hearts with equivalent proportions of diploid and polyploid cardiomyocytes failed to regenerate, demonstrating a need to maintain a diploid state for cardiomyocyte proliferation and myocardial regeneration (Gonzalez-Rosa et al., 2018).

The epicardium, the outermost cell layer of the heart, is reactivated in the regenerating zebrafish heart, characterised by re-expression of developmental genes, such as *Wilms tumour 1* (*wt1*), *raldh2* and *tbx18*, and rapid proliferation (Gonzalez-Rosa et al., 2011; Lepilina et al., 2006). Activated epicardial cells stimulate cardiomyocyte proliferation and neovascularisation, via RA and fibroblast growth factor (FGF) signalling, respectively, and blockade of these signals inhibits regeneration (Kikuchi et al., 2011; Lepilina et al., 2006). Moreover, genetic depletion of the epicardium results in inhibition of cardiomyocyte proliferation and delayed regeneration, highlighting the importance of the epicardium in the regenerative response (Wang et al., 2015). Later work by the same group demonstrated that the pro-angiogenic *vascular endothelial growth factor Aa* (*vegfaa*) was expressed in the adult zebrafish epicardium during homeostasis, and that, following injury, the cells adjacent to the regenerating muscle exhibited both epicardial and endocardial gene expression programmes (Karra et al., 2018). Overexpression of *vegfaa* in the uninjured adult zebrafish heart led to coronary vasculature expansion and an increase in ventricular myocardial wall thickness (Fig. 3). However, whilst ectopic overexpression of *vegfaa* led to global cardiomyogenesis following injury, regeneration at the site of injury was inhibited, thus demonstrating the importance of spatiotemporal control of pro-regenerative factors (Karra et al., 2018).

Studies in zebrafish have emphasised the importance of pre-existing cardiomyocytes in generating new cardiac muscle, as opposed to a cardiac progenitor population, and have highlighted the significance of non-myocyte populations, such as the epicardium and endocardium, in the regenerative response. However, the fundamental differences in the anatomical structure and physiology of the zebrafish and mammalian hearts cannot be ignored. The pressure in a zebrafish heart is low, hence the feasibility of the resection model of injury, whilst an adult mammalian heart is a high-pressured haemodynamic system, meaning resection would result in acute bleeding and rapid death (Hu et al., 2001; Matrone et al., 2017). Zebrafish have a more primitive two-chambered heart, resulting in the mixing of arterial and venous blood. Further, they reside in an aquatic environment, which is substantially more hypoxic. Interestingly, hypoxia has been shown to promote cardiac regeneration in zebrafish by inducing cardiomyocyte dedifferentiation and proliferation (Jopling et al., 2012). Moreover, zebrafish are ectothermic, meaning they rely on external heat sources to regulate their body temperature, enabling them to operate at lower metabolic rates. Conversely, endotherms, such as mammals, predominantly rely on heat generated from internal metabolic processes. Recently, Hirose and colleagues observed an inverse correlation between cardiac regenerative potential and standard metabolic rate, and exogenous injection of thyroid hormone, proposed to induce the ectothermy-to-endothermy transition, resulted in a 45% reduction in cardiomyocyte proliferation and impairment of regeneration in zebrafish (Hirose et al., 2019; Little and Seebacher, 2014). This could suggest that the evolutionary development of endothermy may have led to the loss of cardiac regeneration in higher vertebrates.

Since zebrafish are ectothermic, they need to be able to adapt to changes in external temperature. As such, they are capable of substantial cardiac remodelling, providing modifications in, for example, electrical activity, energy utilisation and structural properties, in response to changes in temperature, thus demonstrating the plasticity of the zebrafish heart (Keen et al., 2017). Further, zebrafish grow continuously throughout life, regulated by factors such as population density, current body size and age (Tsai et al., 2007). Wills et al. observed that, following a decrease in population density, rapid individual animal growth was coupled with cardiac growth, characterised by global cardiomyocyte hyperplasia and an epicardial contribution to the ventricular wall (Wills et al., 2008). Thus, adult zebrafish may be capable of cardiac regeneration because they can utilise mechanisms that maintain cardiac homeostasis and re-purpose the intrinsic adult growth responses following injury.

## Medaka

Medaka (*Oryzias latipes*; Japanese rice fish), like the zebrafish, is a teleost with remarkable capacity for fin regeneration; however, it cannot regenerate its heart (Ito et al., 2014; Katogi et al., 2004). Following resection, a lack of cardiomyocyte proliferation and neovascularisation was observed in the medaka heart, along with a persistent fibrotic scar (Ito et al., 2014). The reasons for divergence in regenerative capacity between these two closely related teleost species remain largely unknown. However, Ito and colleagues reported that medaka do not express endocardial *Raldh2* following injury (Ito et al., 2014). Given this gene's essential role in stimulating cardiomyocyte proliferation in zebrafish (Kikuchi et al., 2011), this may in part cause the lack of regeneration.

When making direct comparisons, the profound physiological differences between fish and mammals may mask important mechanisms for regeneration. Conversely, zebrafish and the non-regenerative medaka are similar anatomically and physiologically, and reside in the same environment (Furutani-Seiki and Wittbrodt, 2004). Moreover, orthologues between these two are more similar than between evolutionarily more divergent species. Therefore, direct comparison between these two species presents a novel approach for deciphering the genes and regulatory pathways essential for cardiac regeneration. To this end, comparative transcriptome analysis between the two species revealed major differences in the immune response. Medaka demonstrated delayed and reduced macrophage recruitment resulting in a persistence of neutrophils compared to zebrafish (Lai et al., 2017), thus implicating an essential role for the immune response in regeneration. Further comparisons between the two fish species are likely to identify other key regulatory pathways in regeneration.

## Astyanax mexicanus

Recently, differences in regenerative capabilities between teleost fish of the same species has been described (Stockdale et al., 2018): *Astyanax mexicanus* (Mexican tetra or blind cavefish) is a single fish species comprising troglomorph (cave-dwelling) and epigean morph (surface) populations in the Sierra de El Abra region of north-eastern Mexico (Gross, 2012). The populations diverged about 1.5 million years ago when surface fish became trapped in at least 29 distinct caves during spring flooding and thus evolved independently (Mitchell et al., 1977). To survive in the dark caves, they developed highly sensitive taste buds and lateral line systems but lost redundant features such as their eyes and pigment (Jeffery, 2009). Of significance, whilst the surface fish population demonstrated complete cardiac regeneration upon ventricular apical resection, a cavefish population from the Pachón cave formed a permanent fibrotic scar. Conversely, fish from both the Tinaja and Chica caves showed substantial variation in regenerative capabilities (Stockdale et al., 2018). This poses the interesting question of whether fish from the Pachón cave lost their ability to regenerate their heart and, if so, what evolutionary competitive advantage have they acquired in doing so?

Interestingly, the hearts of both the regenerating surface fish and the non-regenerating Pachón cave fish displayed significant proliferation of the cardiomyocytes adjacent to the wound area, analogous to the response in zebrafish. Comparative RNA sequencing analysis revealed differences in both the immune and scarring responses, and identified a number of new differentially expressed genes, such as leucine-rich repeat containing 10 (*lrrc10*) (Stockdale et al., 2018). Given the similarities between *A.* *mexicanus* and zebrafish, Stockdale and colleagues genetically knocked out expression of *lrrc10* in zebrafish, and observed impaired cardiac regeneration following cryo-injury (Stockdale et al., 2018), demonstrating how *A.* *mexicanus* can be used to identify essential genes for cardiac regeneration.

Since surface and Pachón cave fish are from the same species, they can inter-breed and produce fertile offspring. Quantitative trait locus (QTL) analysis can then be used to identify loci associated with specific phenotypic changes, enabling the unbiased identification of factors that distinguish between tissue-regeneration-based and scar-based wound healing. Stockdale and colleagues crossed surface fish and Pachón cave fish for two generations, and performed QTL analysis on the resulting F2 generation, revealing a number of newly identified differentially expressed genes, including several that encode for extracellular matrix (ECM) proteins (Stockdale et al., 2018).

Analysing the key genetic, molecular and cellular differences in regenerative capabilities between populations from the same species omits confounders associated with interspecies differences and different injury insults (for example, resection versus cryoinjury), and provides a unique model for studying cardiac regeneration. Further comparative analyses between surface and cave fish populations are likely to identify essential mechanisms for cardiac regeneration.

## Urodele amphibians

Like zebrafish, urodele amphibians, such as newts and axolotls, are able to regenerate several tissues and organs, such as limbs, retina and the heart (Cano-Martinez et al., 2010; Laube et al., 2006; Mitashov, 1997; Simon and Tanaka, 2013). However, long breeding cycles make them difficult to study and their vast genomes, approximately ten times the size of the human’s, complicates sequencing, precluding in-depth molecular and genetic analyses.

### Newts

Studies performed in the 1970s reported limited cardiomyocyte proliferation in the adult newt following apical resection, and incomplete cardiac regeneration up to 30 days post-injury (Becker et al., 1974; Oberpriller and Oberpriller, 1974). However, recent studies have demonstrated complete regeneration 60 days following lateral ventricular resection (Witman et al., 2011) or mechanical crushing (Laube et al., 2006). Cardiac injury led to a substantial downregulation of sarcomere proteins, indicating that cardiomyocyte proliferation originates from the dedifferentiation and proliferation of existing cardiomyocytes (Laube et al., 2006). Piatkowski et al. demonstrated that a transient deposition of ECM components, such as collagen III, preceded the reconstitution of the myocardium (Piatkowski et al., 2013). Additionally, transcriptomic analysis of both newt and zebrafish hearts following apical resection demonstrated that components of the ECM were among the most significantly enriched genes in both, thus demonstrating the importance of the ECM in regeneration and highlighting the conservation of molecular mechanisms in the tissue-regeneration process (Mercer et al., 2013).

### Axolotls

Axolotls are aquatic urodele amphibians capable of cardiac regeneration following both resection and cryo-injury (Cano-Martinez et al., 2010; Lauridsen and Pedersen, 2015). Axolotls are neotenic, meaning that, unlike other amphibians, they do not undergo metamorphosis and retain larval traits into adulthood, remaining aquatic and retaining gills. Under certain circumstances – if they ingest enough iodine, or following thyroid hormone injection – axolotls can metamorphose into a large, land-dwelling adults (Jacobs et al., 1988). To date, no studies have examined heart regeneration in post-metamorphic axolotls. However, studies on limb regeneration revealed a two-fold decrease in regeneration rate and an increase in morphological defects following limb amputation in post-metamorphic axolotls compared to neotenic animals (Monaghan et al., 2014), suggesting that metamorphosis is not conducive to regeneration. It would be interesting to determine whether the post-metamorphic axolotl also has a reduced cardiac regenerative capacity, particularly given the fact that thyroid hormone injection impaired regeneration in zebrafish (Hirose et al., 2019). Furthermore, direct comparison between post-metamorphic and neotenic axolotls could clarify the importance of developmental programmes and morphogenetic events on amphibian regeneration. Whilst differences in cardiac regeneration capabilities have yet to be determined, histological analysis of intact hearts revealed similarities in cardiomyocyte organisation, but the connective tissue surrounded by the muscle fibres was more substantial in the neotenic hearts (Demircan et al., 2016).

### Salamanders

Cardiac regeneration in the salamander, another terrestrial urodele amphibian, has not been previously described. Like the axolotl, they are capable of limb regeneration; however, the process is considerably slower and varies between species (Arenas Gómez et al., 2017). Comparisons between the cardiac regenerative capabilities of aquatic axolotls and terrestrial urodele amphibians, such as the post-metaphoric axolotl and the salamander, could enable the interrogation of the influence of the physiological environment on regenerative response.

## Birds

Unlike fish and amphibians, birds are endothermic animals with a four-chambered heart. A study on chick embryos (*Gallus gallus domesticus*) demonstrated that 3- and 5-day-old embryos were capable of cardiac regeneration following electrothermocoagulation injury (Novikov and Khloponin, 1982). This was characterised by changes in the epicardium and endocardium, and intensive growth of the uninjured myocardium, resulting in complete restoration of the cardiac wall 7-10 days after injury. However, when 18-day-old chick embryos or newly hatched chickens were subjected to the same cardiac injury, the tissue did not regenerate and a scar formed (Novikov and Khloponin, 1982). Cardiac regeneration in chickens, therefore, appears to be restricted to embryonic development, limiting their usefulness as a model to study adult cardiac regeneration.

## Neonatal mice

**Fig. 4.**
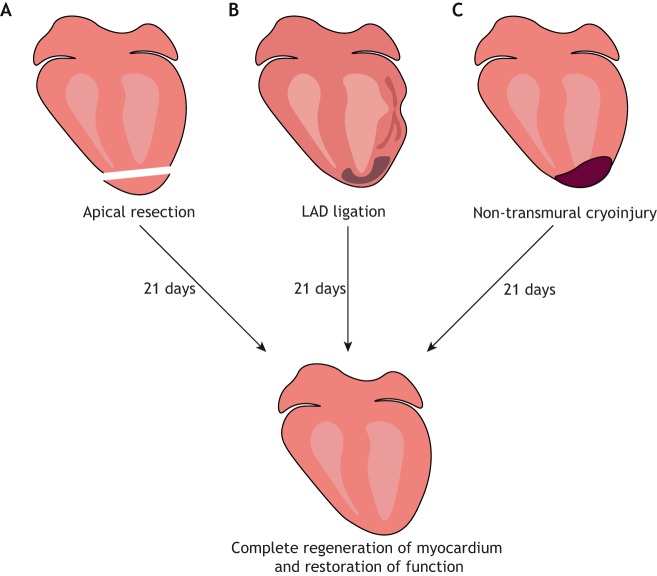
**Mouse models of neonatal cardiac regeneration.** Following a thoracotomy, the heart is exposed from the chest and can be injured by either: (A) removal of the ventricular apex; (B) ligation of the left anterior descending coronary artery (LAD) using a suture; or (C) a cryoprobe held against the ventricle wall.

Whilst the adult mammalian heart has limited regenerative capacity, the neonatal mouse is capable of mounting a substantial response after injury. Pioneering work by Porrello and colleagues demonstrated complete cardiac regeneration in mice following apical resection at postnatal day 1 (P1) (Fig. 4A) (Porrello et al., 2011a). Analogous to the adult zebrafish, neonatal mouse cardiomyocytes undergo hyperplasia to enable normal cardiac growth (Naqvi et al., 2014). Similarly, following injury, genetic fate mapping indicated that the majority of new cardiomyocytes came from the proliferation of pre-existing ones (Porrello et al., 2011a). Therefore, neonatal mice may be capable of cardiac regeneration due to their capacity for cardiac growth. Complete regeneration has also been described following ligation of the left anterior descending (LAD) coronary artery (Fig. 4B) (Porrello et al., 2013), which represents a more clinically relevant model of regeneration, as it more closely recapitulates the ischaemia-induced death of cardiac muscle observed in human MI. Additionally, restoration of cardiac function following non-transmural cryoinjury has been observed (Fig. 4C) (Darehzereshki et al., 2015; Strungs et al., 2013); however, a more severe transmural cryoinjury resulted in incomplete regeneration (Darehzereshki et al., 2015; Jesty et al., 2012), suggesting that a large injury size limits regeneration.

The ability of the neonatal mouse to regenerate its heart has been disputed by some (Andersen et al., 2014, 2016; Zebrowski et al., 2017). Andersen and colleagues observed persistent scarring and dilated cardiomyopathy following apical resection (Andersen et al., 2014, 2016), and reported an increase in cardiomyocyte binucleation, not proliferation (Zebrowski et al., 2017). This discrepancy may be due to differences in surgical technique, since Andersen et al. surgically retracted the ventricle prior to resection, a procedure that has since been shown to cause cardiac injury and persistent fibrosis, even without resection (Bryant et al., 2015). Further, whilst Porrello et al. resected ∼15% of the ventricle, Andersen and colleagues resected more than 30%, and injury size has been shown to be inversely correlated with regenerative ability (Bryant et al., 2015). Despite these inconsistencies, recent longitudinal magnetic resonance imaging (MRI) on mice that underwent LAD ligation at P1 confirmed scar resolution and restoration of function over a period of 21 days (Gunadasa-Rohling et al., 2018). Taken together, these studies revealed, for the first time, that the mammalian heart was capable of regeneration post-birth, thus setting a precedent for reactivating such mechanisms in the adult.

**Fig. 5.**
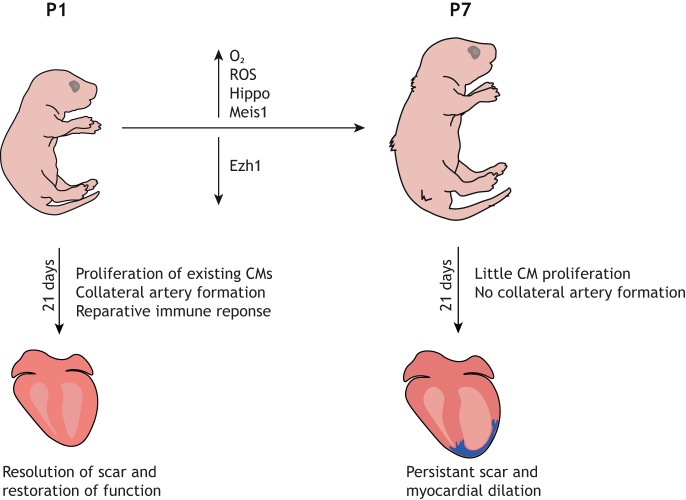
**Loss of cardiac regeneration potential in neonatal mice.** Within the first week of life, the neonatal mouse loses the ability to regenerate its heart. This diagram highlights some of the key changes that occur within the first week of life that contribute to the loss of regenerative capacity. CM, cardiomyocyte; ROS, reactive oxygen species.

The neonatal mouse regenerative capacity, however, is restricted to the first week of life, as P7 mice exhibit a similar fibrotic response to injury to that seen in the adult mouse (Fig. 5) (Porrello et al., 2011a). A recent study demonstrated that this regenerative ability declines rapidly 48 h after birth, with P2 mice also eliciting a fibrotic response (Notari et al., 2018). Whilst the precise duration of the so-called ‘regenerative window’ remains unknown, understanding the molecular and physiological changes that occur shortly after birth that lead to the loss of regenerative potential has been a significant focus of research, with the aim to extend it and eventually invoke regeneration in the adult mammalian heart.

Like the zebrafish, the foetal mammalian heart is fairly hypoxemic due to significant mixing of arterial and venous blood (Dawes, 1955). However, after birth, transition to a postnatal circulation dramatically changes the oxygenation state of cardiomyocytes. The cardiac muscle cells switch from anaerobic glycolytic metabolism to oxygen-dependant mitochondrial oxidative phosphorylation (Fisher et al., 1980; Lopaschuk et al., 1991; Wisneski et al., 1985). This metabolic switch results in a build-up of reactive oxygen species (ROS), which activates the DNA damage response pathway, resulting in a permanent cardiomyocyte cell cycle arrest (Puente et al., 2014). Significantly, postnatal hypoxemia, ROS scavenging or inhibition of the DNA damage response pathway all prolonged the proliferative capacity of cardiomyocytes, resulting in an improved regenerative response beyond the first week of life (Puente et al., 2014). This suggests that changes in the oxygen environment after birth result in permanent cardiomyocyte cell cycle arrest, thus contributing to the loss of regenerative capacity. Oxygen levels were shown to be essential for regeneration in zebrafish (discussed above), thus highlighting how studies in the zebrafish can inform those in the mammal and point to evolutionarily conserved mechanisms of regeneration.

Several studies have examined the signalling pathways and regulatory mechanisms that govern cardiomyocyte proliferation in the neonate. Mahmoud et al. identified the homeodomain transcription factor Meis1 as an essential cardiomyocyte regulator, required for the transcriptional activation of cyclin-dependent kinase inhibitors p15, p16 and p21 (Mahmoud et al., 2013). Heallen and colleagues observed a substantial increase in Hippo activity, measured by levels of phosphorylated Yes-associated protein (pYAP), in mice from P2 to P10 (Heallen et al., 2013). Further, when they performed apical resection on Hippo-deficient mice at P8, they observed increased cardiomyocyte proliferation and functional recovery, suggesting that Hippo signalling is not conducive to regeneration (Heallen et al., 2013). The same group later identified *Pitx2* upregulation in Hippo-deficient regenerating hearts. Pitx2 interacted with YAP and promoted regeneration, in part through inhibition of ROS (Tao et al., 2016). Ai et al. identified EZH1, a component of the epigenetic regulator polyclomb repressive complex 2 (PRC2), as being essential for neonatal cardiac regeneration, through activation of genes related to cardiac growth (Ai et al., 2017). A cross-species transcriptomic screen of three models of regeneration – zebrafish, axolotl and neonatal mice – revealed upregulation of complement 5a receptor 1 (*C5ar1*) in regenerating hearts, and genetic loss of *C5ar1* reduced cardiomyocyte proliferation in the neonatal mouse, thereby identifying activation of *C5ar1* as an evolutionarily conserved response to regeneration (Natarajan et al., 2018).

Along with replacement of lost cardiomyocytes, complete regeneration requires reestablishment of adequate blood flow to the regenerating tissue. This is further substantiated by recent observations by Red-Horse et al., who reported a mechanism by which neonatal mice build collateral arteries in response to injury (Das et al., 2019). Activation of the chemokine CXCL12 in capillary endothelial cells stimulated migration and expansion of arterial endothelial cells along the capillary network, which then reassembled into collateral arteries. Artery reassembly was virtually absent from injured adult hearts but could be induced by exogenous CXCL12 (Das et al., 2019). In addition to differences in neovascularisation, Aurora and colleagues observed differences in the post-MI immune response of P1 compared to P14 mice, and utilised a clodronate-liposome depletion model to demonstrate that monocytes/macrophages are essential for neonatal heart regeneration (Aurora et al., 2014). Macrophages at P1 were transcriptionally similar to the alternatively activated M2 macrophage subclass, and were potentially pro-angiogenic, indicating a putative mechanism by which they might facilitate heart regeneration (Aurora et al., 2014). Additional studies are required to directly compare the immune responses of neonatal versus adult stages, and to dissect out the role(s) of key immune cell types in the regenerative versus pro-fibrotic response to heart injury.

## Neonatal pigs

Regeneration of the neonatal porcine heart was described recently (Ye et al., 2018). Two-day-old pigs subjected to permanent LAD ligation generated new cardiac muscle, recovered function and did not develop fibrosis. However, 14-day-old pigs subjected to the same injury developed extensive fibrotic scars, thinned myocardium and cardiac dysfunction (Ye et al., 2018). This study suggests that the neonatal regenerative capacity extends to large mammals and represents a model that is more anatomically and physiologically similar to the human heart. A caveat to pigs as a model of regeneration is the expense of studies and the paucity of tools for genetic manipulation. Therefore, models such as zebrafish and mice will likely remain popular for underpinning the molecular mechanisms that govern regeneration. Conversely, pig studies may be re-employed at later stages as possible treatments progress closer to the clinic. For example, Gabisonia et al. recently delivered human microRNA-199a (miR-199a) to infarcted pig hearts, which stimulated cardiomyocyte dedifferentiation and proliferation, leading to a marked improvement in global and regional contractility, increased muscle mass and reduced scar size, thus demonstrating that reactivation of endogenous cardiomyocyte proliferation is possible in large animals (Gabisonia et al., 2019). However, persistent, uncontrolled expression of the microRNA led to sudden arrhythmic death, highlighting the need to test such therapies rigorously in large animals to elicit safe and controlled cardiomyocyte proliferation before testing in humans.

## Human neonates

It remains unclear whether, immediately after birth, humans possess equivalent regenerative capacity as mice and pigs. However, there is some anecdotal evidence from clinical case studies to suggest that this may be the case. In 1997, a new-born infant suffered massive cardiogenic shock after a neonatal MI, but her myocardial function recovered completely (Saker et al., 1997). Similarly, in 2015, a new-born child presented with a severe MI following thrombolytic occlusion of the proximal LAD. Although the echocardiogram and biomarkers suggested significant damage to the myocardium, functional recovery was observed within weeks of the initial injury and normal heart function was restored at 1 year of age (Haubner et al., 2016). In addition, children with the rare congenital heart disease anomalous left coronary artery from the pulmonary artery (ALCAPA) showed little or no residual scarring after corrective cardiac surgery (Fratz et al., 2011). Further, studies revealed that cardiomyocyte proliferation contributes towards cardiac growth in young humans, and cardiomyocyte turnover is highest in early childhood (Bergmann et al., 2015; Mollova et al., 2013). Taken together, these reports suggest that humans are capable of cardiac regeneration in early life, and that the molecular and cellular mechanisms that enable such processes in mammalian model systems such as mice are likely to be applicable to patients with ischaemic heart disease.

## Adult mammals

Unlike the above models, the adult mammalian heart has limited regenerative capabilities. Bergmann and colleagues used a carbon-dating technique to demonstrate that, under homeostasis, cardiomyocytes in the adult human heart turn over at a rate of 1% annually. This decreases to 0.45% at the age of 75, and fewer than 50% of all cardiomyocytes are replaced during a normal lifespan (Bergmann et al., 2009). However, it is important to note that this technique infers cellular proliferation via incorporation of C^14^ into genomic DNA; therefore, it cannot be ruled out that an increase in polyploidisation could be confounding the results (Bergmann and Jovinge, 2014). Genetic fate-mapping studies in mice have demonstrated similar turnover levels in the adult mouse and showed that new cardiomyocytes are derived from the division of pre-existing cardiomyocytes and not from a progenitor population (Senyo et al., 2013). Further, following MI, there was a four-fold increase in cell divisions of cardiomyocytes adjacent to the injury, demonstrating that proliferation is not sufficient to elicit complete regeneration of the muscle, but sets a precedent for augmenting the proliferative response to improve regeneration (Senyo et al., 2013).

## Significance for regenerative medicine

The study of regenerative models such as the zebrafish, urodele amphibians and neonatal mice has enabled us to understand some of the intrinsic mechanisms that underpin cardiac regeneration and are essential in stimulating regeneration in the adult. Arguably, one of the most important findings from studies in zebrafish, and more recently the neonatal mouse, is that the majority of new cardiomyocytes in a regenerative model are derived from pre-existing cardiomyocytes as opposed to a cardiac progenitor cell population. Additionally, genetics approaches have provided strong evidence that the adult mammalian heart lacks a true cardiac stem cell population (van Berlo et al., 2014; Li et al., 2018; Sultana et al., 2015). This has shifted the therapeutic focus towards stimulating proliferation of existing cardiomyocytes in the adult mammalian heart following MI. For example, Porrello and colleagues targeted the miR-15 family of microRNAs, which regulate post-mitotic arrest of cardiomyocytes after birth (Porrello et al., 2011b), and revealed that inhibition of miR-15 family members increased proliferation of pre-existing adult cardiomyocytes post-MI and subsequently improved left-ventricular systolic function (Porrello et al., 2013).

A common modality seen across all these regenerative models is the reactivation of developmental gene expression programmes and signalling pathways. For example, several studies in zebrafish have demonstrated that embryonic epicardial genes are re-expressed following injury and are essential for regeneration (Kikuchi et al., 2011; Lepilina et al., 2006; Wang et al., 2015). In the adult mouse, re-expression of developmental gene programmes partially re-activates the epicardium following injury (Smart et al., 2011; Vieira et al., 2017; van Wijk et al., 2012; Zhou et al., 2011), although the response is insufficient to elicit an adequate regenerative response. Zhou and colleagues demonstrated that injecting epicardium-derived cell (EPDC)-conditioned media post-MI decreased infarct size and improved cardiac function (Zhou et al., 2011). Additionally, stimulation of the adult epicardium with the actin-monomer-binding protein thymosin beta-4 (Tβ4) before MI increased mobilisation of epicardial cells post-injury, acting downstream of enhanced activation of epicardial *Wt1*, and favouring neovascularisation and *de novo* generation of cardiomyocytes from EPDCs (Smart et al., 2007, 2011; Vieira et al., 2017). However, when Tβ4 was administered post-MI, it did not affect EPDC fate reprogramming towards cardiomyocytes, nor did it improve the regenerative response (Zhou et al., 2012), presenting a limitation for Tβ4 administration as a treatment following MI. Follistatin-like 1 (FSTL1) was identified as a potent cardiogenic factor secreted by epicardial cells, and application of human FSTL1 via an epicardial patch in mouse and swine models of MI stimulated cardiomyocyte proliferation, leading to an improvement in cardiac function and survival (Wei et al., 2015). However, therapeutic use in human patients has yet to be reported.

Studies in zebrafish and neonatal mice have demonstrated the importance of oxygen levels in cardiomyocyte proliferation and regeneration. Nakada and colleagues subjected adult mice to severe hypoxia following MI. This induced a regenerative response, including a significant increase in cardiomyocyte proliferation, resulting in a decrease in fibrotic scarring and an improvement in cardiac function (Nakada et al., 2017). However, mice were exposed to 7% oxygen, which is substantial hypoxia, and continued exposure increased mortality, presenting a significant caveat to hypoxia as a therapeutic approach.

Comparative analysis between zebrafish and newts demonstrated that components of the ECM, such as tenascin-C, hyaluronic acid and fibronectin, are enriched during regeneration (Mercer et al., 2013). When the ECM protein periostin was delivered to the heart post-MI in both adult mice and pigs, researchers observed an improvement in regeneration (Kuhn et al., 2007; Ladage et al., 2013). More recently, Bassat and colleagues identified the ECM protein agrin as an essential component of neonatal cardiac regeneration in mice, and observed an improved regenerative response in the adult mouse when agrin was administered post-MI (Bassat et al., 2017).

Despite the fact that knowledge gained from these models of regeneration has led to promising improvements in regeneration in adult mice, no meaningful drugs or treatments have thus far been translated to the human patient. This may reflect inherent differences between the mouse and human heart, demonstrating the need to replicate findings in a larger mammalian model such as the pig. Importantly, some of these studies in mice demonstrated an improvement in regeneration using genetic manipulation (Porrello et al., 2013) or, in the case of the Nakada study, a life-threatening low-oxygen environment (Nakada et al., 2017). Whilst these have provided important insight into the signals that govern regeneration, they are not translatable into the clinical setting. As such, an unmet clinical need to identify novel therapeutics for targeting pro-regenerative pathways without genetic or environmental manipulation remains. Finally, the study of these model organisms of regeneration has demonstrated that regeneration does not occur solely from the proliferation of one population of cells but rather from the cooperation of many distinct cell types, in concert with modulation of the local inflammatory and fibrotic milieu, leading to removal of the injured tissue, transient scar formation, generation of new cardiac muscle, and neovascularisation. Therefore, it is likely that we will need to stimulate such a coordinated response in human MI patients, utilising combined therapies to elicit a substantial improvement in regeneration.
